# Findings and lessons learned from developing a 5-year community-based intervention for preventing early marriage in rural Gambia

**DOI:** 10.1186/s12978-025-01992-5

**Published:** 2025-05-31

**Authors:** Mat Lowe, Mariama Bojang, Alhagie Gaye, Isatou Njie, Awa Dubois

**Affiliations:** Society for the Study of Women’S Health (SSWH), Kanifing, The Gambia

**Keywords:** Early marriage, Gambia, Rural, Adolescents, Boys, Girls

## Abstract

**Background:**

Preventing Early Marriage in Rural Gambia: Testing an Intervention was a 5-year project that aimed to address early marriage among girls in 53 rural communities in The Gambia. At baseline, the aim of the project was to identify the social and cultural factors that contribute to early marriage for girls aged 10–19. The baseline findings revealed that factors such as ethnicity and parents' concerns about their daughters engaging in premarital sex were significant contributors to early marriage for girls. Additionally, the lack of viable alternatives to marriage was also identified as a key factor. This information was utilized by the project team to design and implement the project intervention that included community engagement forums and discussion sessions and capacity building for key community stakeholders.

**Methods:**

This study compared the project's baseline and endline data to assess the impact of the project intervention on girls' age at first marriage and changes in parents' knowledge of and attitudes toward early marriage and its prevention. It utilized a non-experimental evaluation design.

**Results:**

The study results showed a significant increase in the average age of girls at first marriage, from 15.9 at baseline to 23.9 years at endline (*P* < 0.0001). Additionally, parents who actively participated in the community engagement forums and discussions have significantly improved their understanding of the harmful effects of early marriage on girls. This new knowledge has empowered these parents to re-evaluate the necessity of early marriage for girls.

**Conclusion:**

A key lesson we learned from developing this project intervention is that locally-based interventions, carefully designed and implemented with meaningful participation from key community stakeholders, have the potential to address the underlying causes of early marriage for girls in rural communities in The Gambia.

## Plain English

Early marriage, also defined as marriage below the age of 18, mainly affects girls. It is common in many West African countries, including The Gambia. This study sought to document the findings and lessons learned from developing a five-year community-based intervention for addressing early marriage among girls aged 10–19 in 53 rural communities in The Gambia.

## Résumé

**Contexte:**

*Prévention du mariage précoce en Gambie rurale: expérimentation d’une intervention* était un projet de cinq ans qui visait à lutter contre le mariage précoce des filles dans 53 communautés rurales de Gambie. Au départ, l’objectif du projet était d’identifier les facteurs sociaux et culturels qui contribuent au mariage précoce des filles âgées de 10 à 19 ans. Les constatations de base ont révélé que des facteurs tels que l’ethnicité et les inquiétudes des parents concernant les relations sexuelles de leurs filles avant le mariage contribuaient grandement au mariage précoce des filles. En outre, le manque d’alternatives viables au mariage a également été identifié comme un facteur clé. L’équipe du projet a utilisé ces informations pour concevoir et mettre en œuvre l’intervention du projet qui comprenait des forums de mobilisation communautaire et des séances de discussion ainsi que le renforcement des capacités des principales parties prenantes de la communauté.

**Méthodes:**

Cette étude a comparé les données de base et finales du projet pour évaluer l’impact de l’intervention du projet sur l’âge des filles au premier mariage ainsi que l’évolution des connaissances et des attitudes des parents à l’égard du mariage précoce et de sa prévention. Un modèle d’évaluation non expérimental a été utilisé dans le cadre de cette étude.

**Résultats:**

Les résultats de l’étude ont montré une augmentation significative de l’âge moyen des filles au premier mariage, de 15,9 ans au départ à 23,9 ans à la fin (*P* < 0,0001). En outre, les parents qui ont participé activement aux forums et aux discussions de mobilisation communautaire ont considérablement amélioré leur compréhension des effets néfastes du mariage précoce sur les filles. Ces nouvelles connaissances ont permis à ces parents de réévaluer la nécessité du mariage précoce pour les filles.

**Conclusion:**

L’une des principales leçons que nous avons tirées de l’élaboration de cette intervention de projet est que les interventions locales, soigneusement conçues et mises en œuvre avec la participation significative des principaux intervenants de la communauté, ont le potentiel de s’attaquer aux causes sous-jacentes du mariage précoce des filles dans les communautés rurales de Gambie.

## Background

Early marriage, also known as child marriage, is the practice of getting married before the age of 18. This practice mainly affects girls and is widely recognized as a serious violation of human rights by international agreements such as the Convention on the Rights of the Child and the Convention on the Elimination of All Forms of Discrimination against Women [1, 2]. Early marriage is a global problem and is addressed in the Sustainable Development Goals (SDGs), specifically in SDG 5 on gender equality, target 5.3. However, despite this global recognition, early marriage continues to be a global issue.

In West and Central Africa, the rate of decline in early marriage has been the slowest [3, 4]. This region has the highest prevalence of child marriage, with estimates varying from (76%) in Niger to (18%) in Cape Verde [5]. Four in ten girls in West and Central Africa marry before the age of eighteen [6], and one in three marry before the age of 15. Due to the growing population of girls in the region, the number of child brides in West and Central Africa is projected to increase from 6.4 million in 2015 to 7.1 million by 2030 [4]. In this region, girls often marry older spouses with multiple partners in a polygamous setting [7]. This makes it difficult for the girls to effectively negotiate safer sex, leaving them vulnerable to sexually transmitted infections (STIs) including HIV/AIDS [8–10]. The sub-region also has the highest rates of early motherhood in the world [11]. Intense pressure on girls to conceive soon after marriage leads to early pregnancies, short birth spacing, and a higher number of children [6]. In the region, in 2009, approximately 13.4 percent of women aged 20–24 years gave birth before the age of 16 and nearly 31 percent by the age of 18 [9]. The link between too early childbearing and increased risk of maternal and new born health problems remain unclear. However, it is suggested that biological, behavioral, social, and economic factors combine with inadequate access to and use of health services that could exacerbate health problems, directly raising the risk of maternal and new born health problems [9–12]. The physical immaturity of young girls leads to negative health outcomes that contribute to high maternal and neonatal mortality in Sub-Saharan Africa. Early childbearing is associated with maternal health problems including obesity, anemia, malaria, sexually transmitted infections, mental illness, and obstetric fistula [9, 13, 14]. Adolescent mothers' infants also have a higher risk of mortality, stillbirth, low birth weight, and premature birth [11]. Early marriage also increases the burden of pregnancy-related death and illness for women [9–11].

Young married girls often face barriers to entering the labor market due to early and frequent childbearing, large family sizes, and limited access to education [15–21]. This limits their formal employment opportunities and often confines them to household chores or informal sector jobs. The impact of early marriage on labor force participation varies by country or community, and it can lead to household poverty and hinder economic growth. This is particularly evident in West and Central African countries. The reasons for the prevalence of early marriage in West and Central Africa are diverse and can be categorized under religion, tradition, culture, poverty, and gender inequalities [10, 22, 23].

In The Gambia, although the practice of early marriage has decreased from (58% to 30%) over the past two decades, it is prevalent [24]. The reasons for the persistence of early marriage in The Gambia are varied and include the fear of unwanted pregnancy, the protection of family honor, poverty, societal pressures from close relatives and community members, and the continued demand from older men for younger brides, culture, lack of meaningful opportunities, and the inadequate enforcement of the law banning child marriage [25]. These factors have continued to perpetuate the practice of early marriage in The Gambia. To help further accelerate the decline in early marriages among girls in The Gambia, the Society for the Study of Women’s Health (SSWH) and the Agency for the Development of Women and Children (ADWAC) implemented the project, ‘Preventing Early Marriage in Rural Gambia: Testing an Intervention’.

### Project description

Preventing Early Marriage in Rural Gambia: Testing an Intervention was implemented from October 2018 to October 2023 in 53 rural communities across two districts—Lower and Central Baddibu Districts in the North Bank Region of The Gambia. In the initial exploratory phase, the goal of the project was to uncover the social and cultural factors contributing to early marriage among girls aged 10–19 years. The exploratory study utilized a mixed-methods approach that included collecting quantitative data through a cross-sectional household survey with a sample of 181 female and 169 male adolescents, as well as qualitative data through focus group discussions with 16 male and female parents, and eight key informant interviews with three female and five male community-based decision makers who were conveniently selected from Lower Baddibu District in the North Bank Region of The Gambia. The study found that ethnicity and parents’ fear that girls may engage in premarital sex are two important factors associated with early marriage for girls. Other factors included lack of meaningful alternatives to marriage, including work opportunities in rural areas, which may also limit the options and resources available to girls, resulting in early wedlock [25]. These findings were utilized to guide the co-creation and design of the intervention components of the project with community-based committees and local steering committee of the project. The project intervention components included community engagement forums and discussion sessions, capacity building for key community stakeholders, awareness raising sessions and educational workshops for adolescent girls and boys, livelihood skills training for adolescent girls for improved economic development opportunities, and support for girls to remain in school (Table 1).

**Table 1:** Baseline research findings and intervention design

Research findings	Intervention component
Ethnic values of early marriage	Community engagement forums and discussion sessions to collectively explore ethnic differences in marriage patterns and stimulate discussion around the ethnic values of early marriage
	Capacity building for key community stakeholders to form a ‘core communication’ team for social mobilization and door to campaign on prevention of early marriage
Fear of premarital sex	Awareness raising sessions to allay parents’ fears about premarital sex and training workshops on sexual and reproductive health for adolescent girls and boys
Lack of meaningful alternatives to marriage, including work opportunities and resources	Economic development opportunities provided for girls through training on tie and dye, soap making and hand-sewing
	Support girls to remain in school to address economic barriers to schooling and reduce the risk of school dropout and subsequent early marriage

The project aimed to achieve two key outcomes through the implementation of the various intervention components. One goal was to reduce early marriage by increasing the age at which girls get married. The other goal was to increase knowledge about early marriage and its prevention at the individual and community levels. The various intervention components are outlined below.

### Community engagement forums and discussion sessions

Community engagement forums and discussion sessions are widely used in The Gambia to sensitize and transform gender and social norms. They provide a platform for community members to participate in mutual learning and develop decisions on addressing societal issues while preserving trust and accountability.

The project organized community engagement forums and discussion sessions throughout the project lifespan. The community engagement forums and discussion sessions were open to all members of the communities in the two project implementation districts (Lower and Central Baddibu Districts). The project field coordinators held these forums and discussion sessions once every month. The purpose was to collectively explore ethnic differences in marriage patterns and stimulate discussions around ethnic values on early marriage, aimed at promoting gender and social norms change in marriage patterns. The community engagement forums and discussion sessions were delivered through movie screenings to shift social and gender norms around early marriage. Additionally, the project engaged young men as advocates of change to organize community engagement forums and discussion sessions, aimed at dispelling gender and social norms around early marriage and male dominance. This was achieved through training their peers, drama, and role play at the community level.

### Capacity building for key community stakeholders

The project organized interactive training workshops for the community-based committees established by the project. Two hundred community-based committee members, including both male and female members, attended the workshops. The topics discussed during the workshops included traditional gendered practices such as early marriage for girls, and the role of men in preventing harmful gendered practices like early marriage. Following the capacity building workshops, the project supported the members of the community-based committees in forming a 'core communication' team for social mobilization and door-to-door campaigns on preventing early marriage in their respective communities.

### Awareness raising sessions and educational workshops for adolescents

Awareness-raising sessions were organized, mainly targeting parents. The objective of these sessions was to alleviate parents' fears about premarital sex. The sessions were held once a month and were open to parents in all project intervention communities. In addition, young adolescent girls and boys were trained on sexual and reproductive health knowledge, including how to prevent teen pregnancy and avoid early sexual debut. The training covered topics such as building skills to negotiate for condom use and avoid early sexual intercourse. A total of 200 boys and 250 girls attended these trainings. The trainings were delivered by field coordinators, and later, trained peer educators, who are young boys and girls themselves and whose knowledge is rooted in community culture, norms, and traditions, conducted step-down trainings for their peers.

### Support girls to remain in school

It is well-documented that keeping girls in school will contribute to delaying their marriage [26]. Based on this understanding and the baseline finding, the project provided educational support to six girls who were at risk of school dropout and early marriage due to their family vulnerability. The support provided to these girls covered school costs, including uniforms, bags, shoes, and financial assistance for school-related expenses.

### Livelihood skills training to enhance the economic development opportunities of girls

Girls were provided with employment-oriented opportunities, including vocational skills training, to address their lack of meaningful alternatives to early marriage and to boost their economic development opportunities. The training enhanced the livelihood skills of forty-nine girls through instruction in soap making, tie and dye, and hand-sewing for income generation.

**Fig. 1:**
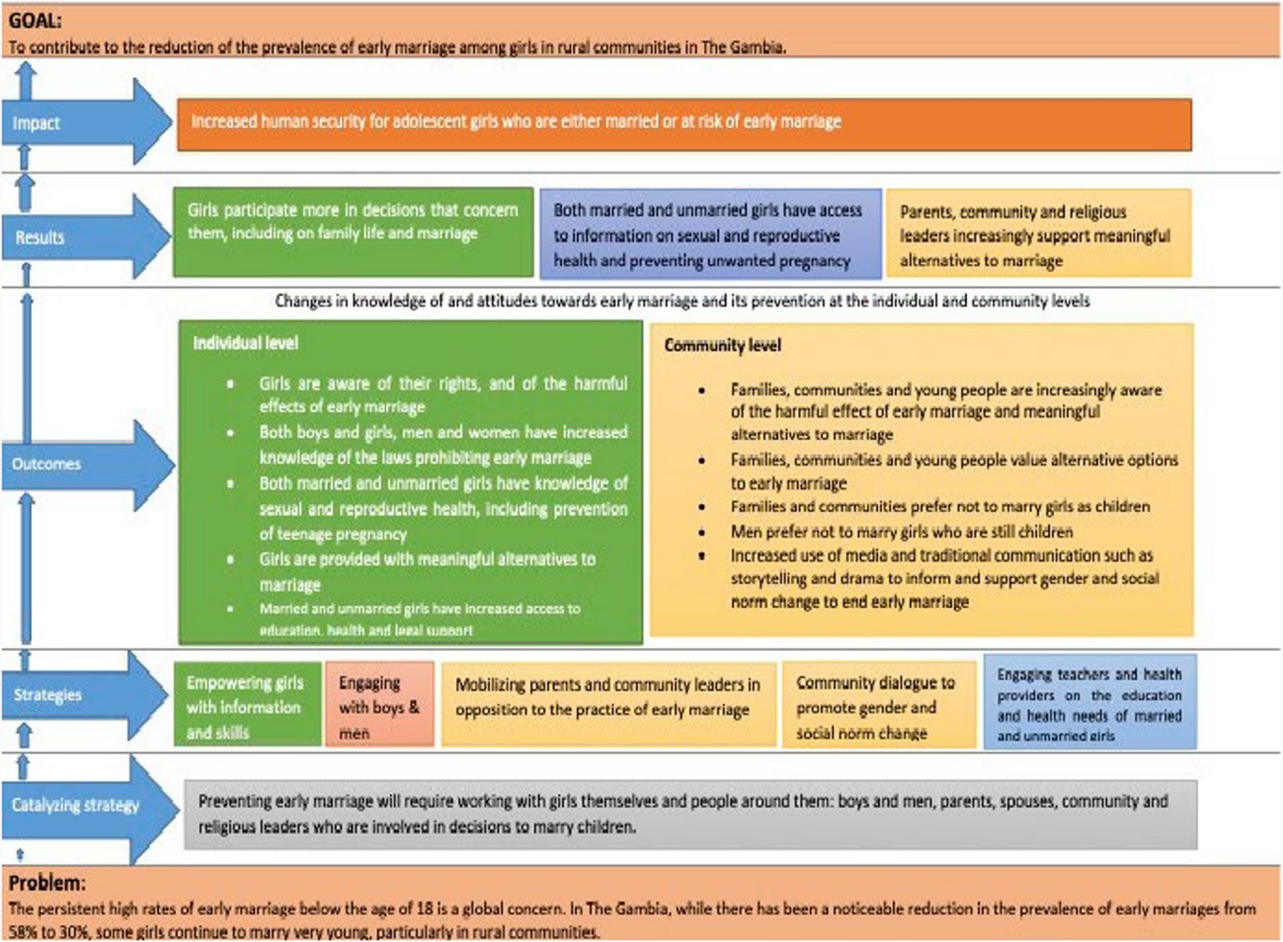
Project theory of change

The thinking behind the specific set of intervention components implemented was based on the project’s baseline data and findings, which revealed how ethnicity, parents’ fears about premarital sex, and limited meaningful alternatives, including work opportunities in rural areas can influence early marriage for girls. The implementation of the specific set of intervention components was also based on the project Theory of Change (ToC) that was developed (Fig. 1). The project ToC has as a goal to contribute to the reduction of the prevalence of early marriage among girls in The Gambia. It highlights the decline but persistence of early marriage in The Gambia as a major problem to address and argues that to achieve the goal of eradicating it completely, a broader catalyzing strategy should be adopted. This strategy should recognize that early marriage cannot be addressed in isolation and should involve working with girls themselves, as well as the people around them—boys and men, parents, spouses, community, and religious leaders who are involved in early marriage decisions. The catalyzing strategy should aim to change the social norms around early marriage and empower girls with information on the issue. It should also engage boys and men, mobilize parents and community leaders to oppose the practice of early marriage, and encourage community dialogue to promote social norms change. These strategies are interwoven and mutually reinforcing, and ending early marriage will require a combination of actions related to all these strategies. By implementing these strategies, the project envisioned reduction in early marriage for girls and changes in knowledge of and attitudes towards early marriage and its prevention.

## Methods

### Design

The study used a non-experimental design and included baseline and endline survey data.

### Data collection

**Fig. 2:**
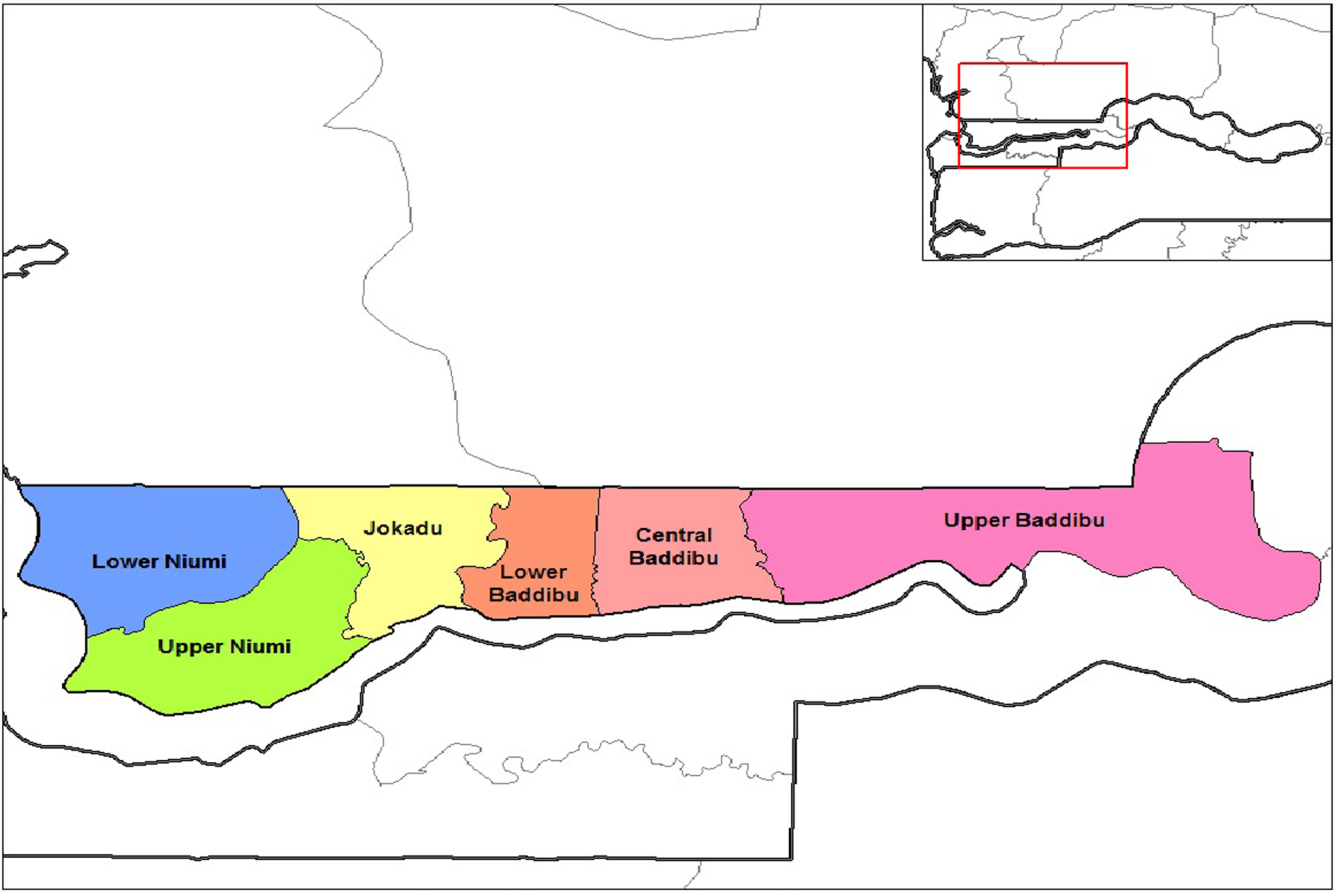
Map of the districts of the North Bank Region, showing Lower and Central Baddibu Districts = . Source: Wikimedia Commons

A baseline survey was conducted in late 2018 in rural communities in Lower and Central Baddibu Districts (Fig. 2). Lower and Central Baddibu Districts were chosen because they have high prevalence of early marriage. Both districts have a median age at first marriage of 17.3 years, which is below the legal minimum age of marriage in The Gambia. Shortly after completion of the baseline survey, implementation of the project began in the two districts. An endline survey was conducted in late 2023 following the implementation of the project in both districts. Similar sample selection and data collection techniques were used for the baseline and endline surveys. All households in the two districts were listed, and demographic information on all household members was collected. Only households with a male or female adolescent aged 10–19 were eligible at baseline; for the endline survey, only households with a female adolescent were included. To assess the impact of the project on girls, the baseline data for male adolescents were dropped from the analysis. Female adolescents (aged 10–19 years) were the main focus of the endline survey because the practice of early marriage has a greater impact towards young females. The number of female adolescents selected from each community in the two districts was proportionate to the size of the population.

Parents of adolescent females were also selected and interviewed during the endline survey. The data collectors selected households of female adolescents having either one of the parents (male or female, who were self-identified). One parent was interviewed from each selected household.

Eight data collectors recruited administered the surveys; minimum qualifications included prior interviewing experience and a secondary school education. The project used data collectors who were relatively young (20–28) to make adolescents more comfortable and responsive for the surveys with adolescent males and females. However, for the baseline and endline surveys with parents, data collectors were much older to also make parents more comfortable and responsive. Data collectors received one day of training prior to the initial household listing and an additional five days of training before conducting surveys. During the training, they reviewed each item on the survey and engaged in one-on-one and group practice interviews. Once data collection began, they made up to three attempts to locate and interview selected adolescents and parents.

Because of the sensitive nature of the survey topics, adolescents and parents were interviewed by data collectors of the same gender. The interviews generally took place at the household of adolescents and parents, during which interviewers ensured auditory privacy.

The study primarily used closed-ended questions to gather data on the demographic characteristics of the respondents, age at marriage, their perspectives on the suitable age for marriage, reasons for early marriage, and decision-making roles related to early marriage. Both the baseline and endline surveys were the same for female adolescents. However, for the survey with parents, they were mainly asked about their socio-demographic characteristics and participation in the project intervention components, particularly the community engagement forums and discussion sessions. The questionnaires were pre-tested to allow for revisions and finalization before full-scale administration at baseline. The parent questionnaire was also pretested before being used at endline.

### Measures

At both baseline and endline surveys, respondents were asked if they had ever attended school and if they were currently attending school. They were also asked how many years of schooling they had received, and how well they could read and write. Marital status was determined by asking if they had ever been married. To determine whether the marriage of girls declined during the project period, we constructed a variable to reflect recent marriages; by comparing a respondent’s current age and age at first marriage, we determined whether she had gotten married in the prior year [26]. We then compared the proportions of girls married in the year prior to both the baseline and the endline surveys; the latter marriages occurred during the period in which the project was being implemented in the two Districts [26]. We evaluated the impact of the implementation of the project intervention by surveying parents to understand their participation in the various components of the project intervention, notably the community engagement forums and discussion sessions and awareness-raising events. We compared parents who reported participation with those who did not, to see if their involvement in community engagement forums and discussion sessions, and awareness-raising events increased their knowledge of the harmful effects of early marriage on girls. We also looked at whether the knowledge gained through their participation had an impact on their perceptions and decision to reconsider the practice of early marriage for girls.

### Data analysis

Descriptive analysis of respondents’ demographic characteristics was first conducted using the means and standard deviations for continuous variables and the frequencies and percentages for categorical variables. Paired T-test was used for comparing responses in baseline and endline. The data analysis also involves both descriptive and inferential statistics to understand and detect significant changes in the variables measured in both waves. The analysis was limited to female adolescents who participated in both waves and for parents who participated in the endline survey. It was only done at the individual level. All the data were analyzed in Stata 17.0.

## Results

### Sample characteristics

Table 2 shows the sample characteristics of female adolescents in both waves. The average age at baseline and endline was similar (16.2 vs. 16.0 years) with a *P*-value of 0.75, indicating no significant change over time. Almost all participants identified as Muslim at both baseline (98.9%) and endline (97.1%), showing consistency in religious demographics. There were significant changes in the ethnic composition, with the proportion of Fula decreasing (from 40.5 to 25.7%) and Wolof increasing (from 11.4 to 28.8%) from the baseline to the endline (*P*-value < 0.001).

**Table 2:** Sociodemographic characteristics of adolescents in both waves

Variable	Baseline		Midline		P value^1,2^
	Mean (SD)	% n = 76	Mean (SD)	% n = 76	
Age in years at survey time	16.0 (3.72)		16.2 (4.42)		0.75
*Religion*					
Christian		0		0	
Moslem		98.9		97.1	
Traditional		0		0	
*Ethnicity*					
Fula		40.5		25.7	< 0.001
Mandinka		46.8		37.8	
Serer		1.27		7.58	
Wollof		11.4		28.8	

1 = Paired T test for continuous variables; 2 = McNemar test for categorical variables

Table 3 shows the education related variables of female adolescents in both waves. At baseline, (57.1%) could read and write, which slightly decreased to (50.0%) at endline. The change is not statistically significant (*P*-value: 0.08). At baseline, (55.1%) had ever attended school, reducing marginally to (52.86%) at endline but the difference is not statistically significant (P-value: 0.25). There is a notable decrease from (45.4%) at baseline to (34.3%) at endline in current school attendance, which is statistically significant (*P*-value: 0.03). Based on the data, the two groups are comparable in terms of having ever attended school and the ability to read and write, as the differences are not statistically significant. However, there is a significant difference in the proportion of girls currently attending school, indicating a change between the two time points. This suggests that while the groups are comparable on some educational aspects, there has been a decline in school enrolment over time.

**Table 3:** Education related variables of adolescents in both waves

Variable	Baseline % n = 76	Midline % n = 76	P value^1^
*Girl can read and write*			
Yes	57.1	50.0	0.08
*Ever attended school*			
Yes	55.1	52.86	0.25
*Currently attending school*			
Yes	45.4	34.3	0.03

1 = McNemar test for categorical variables

Table 4 shows comparison of socio-demographics characteristics of parents who participated in community sensitization and community-based committee meeting or training workshop and those who do not. In terms of gender, (56%) of the participants were men, while (54.3%) of non-participants were women. The majority of participants and non-participants were aged 32 and older, with (48.4%) and (41.8%) respectively being 45 years and older. Among the participants, (14%) had no formal education, (12.7%) had completed primary school, and (57.3%) had attended informal Arabic school. In contrast, (30.5%) of non-participants had no formal education, and (42.5%) had attended Arabic school. In terms of ethnicity, (31.8%) of participants were Wolof, and (27.4%) were Mandinka. Among non-participants, the main ethnic group was Mandinka, followed by Fula and Wolof. When comparing those who participated in community activities, (55%) were men, while (53.4%) of non-participants were women. These findings suggest a gender discrepancy in participation and indicate the influence of men in decision-making regarding their daughters.

**Table 4:** Comparison of socio-demographics characteristics of parents who participated in awareness raising workshops and community engagement forums and discussion sessions and those who do not

Variable	Has participated in community awareness raising sessions on early marriage		P value (Pearson’s chi square)	Has participated in community engagement forums and discussion sessions on early marriage		P value (Pearson’s chi square
	Yes n = 157 %	No n = 282 %		Yes n = 157 %	No n = 282 %	
*Gender*						
Male	88 (56.0)	129 (45.7)	0.03	82 (55.0)	135 (46.5)	0.09
Female	69 (43.99)	153 (54.3)		67 (44.9)	155 (53.4)	
*Age*						
18–24 years	7 (4.46)	31 (10.9)	0.07	9 (6.04)	29 (10.0)	0.22
25–31 years	29 (18.5)	61 (21.6)		29 (19.5)	61 (21.0)	
32–38 years	45 (28.7)	72 (25.5)		36 (24.2)	81 (27.9)	
45-years or older	76 (48.4)	118 (41.8)		75 (50.3)	119 (41.0)	
*Educational level*						
No schooling completed	22 (14.0)	86 (30.5)	0.006	17 (11.4)	91 (31.4)	< 0.001
Primary school leaving certificate	20 (12.7)	36 (12.7)		19 (12.7)	37 (12.7)	
Higher school or leaving certificate	20 (12.7)	34 (12,1)		19 (12.7)	35 (12.1)	
Arabic school	90 (57.3)	120 (42.5)		85 (57.0)	125 (43.1)	
Certificate /diploma	4 (2.55)	4 (1.42)		7 (4.70)	1 (0.34)	
Bachelor’s degree	1 (0.64)	2 (0.71)		2 (1.34)	1 (0.34)	
*Respondents’ ethnic group*						
Mandinka	43 (27.4)	127 (45.0)	0.002	42 (28.2)	128 (44.1)	0.03
Wolof	50 (31.8)	55 (19.5)		44 (29.5)	61 (21.0)	
Jola	1 (0.64)	2 (0.71)		1 (0.67)	2 (0.69)	
Serer	3 (1.91)	6 (2.13)		2 (1.34)	7 (2.41)	
Sarahule	0	3 (1.06)		0	3 (1.03)	
Fula	45 (28.7)	71 (25.18)		45 (30.2)	71 (24.5)	
Bambara	15 (9.55)	14 (4.96)		14 (9.4)	15 (5.17)	
Other (specify)	0	4 (1.42)		1 (0.67)	3 (1.03)	

Notably, the age groups that have shown the highest levels of participation are those aged 32 years, accounting for (24.2%), and the group of 45 years and older, with a significant participation rate of (50.3%). This trend is mirrored in the groups that did not participate in the activities, underscoring the importance of age in community engagement in The Gambia. One of the key findings is the educational disparity among the participants and non-participants. The majority of both groups have an educational background from Arabic schools. However, it is concerning to note that (11.4%) of participants and a staggering (31.4%) of non-participants have not completed any schooling, indicating a significant educational gap that could potentially affect community engagement.

### Changes in age at first marriage, knowledge of, and attitudes towards early marriage

Table 5 presents data on marriage arrangements and age at first marriage for girls. The results indicate that at baseline, (25%) of girls were aware that their parents had arranged a marriage for them, but this decreased to (15.7%) at endline. The average age at first marriage for girls increased significantly from 15.9 at baseline to 23.9 years at endline (*P* < 0.0001). One of the reasons that could explain the changes in marriage arrangements and increase in average age of marriage for girls includes exposure to the project intervention. For instance, the study results revealed that parents who actively participated in the community engagement forums and discussion sessions organized by the project heard more frequently about the adverse health and social consequences of early marriage on girls. The impact that the knowledge gained has had on the view of early marriage among these parents indicates that it made them re-evaluate early marriage for girls and its necessity (Table 6).

**Table 5:** Marriage arrangement and age at marriage among girls

Variable	Baseline % n = 76	Midline % n = 76	P value
Ever heard that your parents arranged for you to get married?			
Yes	25.0	15.7	0.08
Age when first heard that your parents had arranged for you to get married? Average	15.9	22.2	< 0.001
Age when first got married or lived with a man? Average	15.9	23.9	< 0.0001

**Table 6:** Knowledge and attitudes towards early marriage and participation in community sensitization and community-based committee meeting or training workshops

Variable	Has participated in community sensitization on early marriage		P value (Pearson’s Chi square)	Has participated in Community-based committee (CBC) meeting or training workshop on early marriage		P value (Pearson’s Chi square)
	Yes n = 157 %	No n = 282 %		Yes n = 149 %	No n = 290 %	
*Ever heard about the negative consequences of early marriage*						
Yes	144 (91.7)	225 (79.8)	0.001	134 (89.9)	235 (81.0)	0.016
No	13 (8.28)	57 (20.2)		15 (10.0)	55 (18.0)	
*Would you please indicate the negative consequences of early marriage that you have heard ?*						
High risk pregnancy and childbirth	30.9	27.6	0.008	27.4	28.5	0.022
Physical illness	19.5	19.3		18.8	19.0	
Depression	4.87	5.88		4.60	5.87	
Emotional distress	7.79	6.14		7.82	5.87	
Dissatisfaction with married life	5.34	6.78		5.29	6.63	
Lack of independence in family life	6.57	3.71		5.98	3.83	
Maternal death	17.7	19.9		16.7	19.9	
Divorce	7.3	5.12		6.90	5.10	
Domestic violence	5.95	5.50		6.44	5.23	
*What is the impact of the information you have heard on your view on early marriage?*						
It has made me re-evaluate girls’ early marriage and its necessity	20.3	18.9	0.023	18.1	20.1	0.001

## Discussion

Preventing Early Marriage in Rural Gambia: Testing an Intervention was developed to address early marriage among girls in rural communities in Lower and Central Baddibu Districts in the North Bank Region of The Gambia. It is one of the first projects to design and evaluate interventions on early marriage in The Gambia based on locally generated research findings that fully characterized contextual factors influencing decisions on early marriage in rural Gambian communities.

The project's baseline findings recognized that the reasons behind the high prevalent of early marriage among girls in communities in Lower and Central Baddibu Districts are multifaceted. They include ethnicity, parental concerns about premarital sex, and the absence of viable alternatives to marriage. Drawing from these findings, the project team developed and executed interventions aimed at tackling the influence of ethnicity on marriage patterns, easing parental anxieties about premarital sex, and offering girls education focused on improving their knowledge of sexual and reproductive health and employment opportunities, including vocational skills [25].

This study results showed that the project has contributed to an increase in age at marriage, giving girls critical extra years to expand their social networks, attend school, and develop as individuals [26]. The study also highlighted significant impact on parental involvement in the project intervention components. This involvement has led parents to reconsider the practice of early marriage for girls. However, it was observed that more male parents than female parents participated in the activities of the project, indicating a gender imbalance and suggesting that men have a significant influence on decisions regarding their daughters. This finding confirms the influence of patriarchal norms in marriage decisions, where men/fathers are the primary decision-makers regarding when and to whom a girl gets married [26, 27]. It also highlights the necessity of increasing women's participation in early marriage prevention programs.

The current study adds to the increasing evidence on the effectiveness of multi-component community-based interventions in preventing early marriage for girls. However, the study is limited by several factors that warrant consideration in interpreting the findings. First, it is possible that participants may have responded in a way that is socially desirable during interviews and surveys, which could affect the study's results. The high levels of exposure to all project intervention components also made it difficult to ascertain which component of the project intervention may have been more impactful in contributing to the change in marriage arrangements, age at marriage and knowledge of and attitudes towards early marriage and its prevention as found in the study. Individual analysis of each component of the project on the change (impact) they have had was impossible because it was rare to find one area or community where only one component of the project was implemented. However, this would be a shortcoming of many evaluations of similar research on early marriage because the factors perpetuating early marriage are inextricably linked and no single intervention can address it [28]. Preventing Early Marriage in Rural Gambia: Testing an Intervention simultaneously addresses the economic and social factors that promote early marriage, and provides girls with alternative pathways. Such approaches have tremendous potential in The Gambia and in other West African countries with similar socio-demographics and norms, such as Niger, Mali and Nigeria, where large numbers of girls are married before age 15 [29–31]. The study results on the impact of the Preventing Early Marriage in Rural Gambia: Testing an Intervention project demonstrate that the incentives and traditions that support early marriages can be changed by addressing motivations for arranging marriages for young girls [26]. The study results also align with other intervention studies to prevent early marriage, showing that community dialogues are effective in this regard [32, 33]. It also supports the growing evidence that keeping girls in school and providing them with educational support is crucial for the success of early marriage prevention programs. Education plays a central role in preventing early marriage and is interconnected with other aspects of girls' and adolescents' socio-ecological framework [33, 34]. The effectiveness of community-based interventions to delay early marriage for girls has also been observed in India [35], which is consistent with the results of this study.

In future research, it will be interesting to explore whether the observed increase in the age of marriage for girls could lead to empowerment within marriage for these girls. The study also suggests a need for further exploration of the factors contributing to differences in marriage patterns between ethnic groups, as observed in the baseline survey.

## Conclusion

The study results suggest that interventions designed and implemented with meaningful participation from key community stakeholders, have the potential to address early marriage for girls in rural communities in The Gambia.

## Data Availability

The data used for this study is available at https://doi.org/10.6084/m9.figshare.26827018.v1.

## Abbreviations

ADWAC: Agency for the Development of Women and Children
CB: Central Baddibu
IDRC: International Development Research Centre
LB: Lower Baddibu
SSWH: Society for the Study of Women’s Health
